# Nanoscale Bilayer Mechanical Lithography Using Water as Developer

**DOI:** 10.1021/acs.nanolett.1c00251

**Published:** 2021-04-22

**Authors:** Yu Shu, Benjamin F. Porter, Eugene J. H. Soh, Nikolaos Farmakidis, Seongdong Lim, Yang Lu, Jamie H. Warner, Harish Bhaskaran

**Affiliations:** † Department of Materials, University of Oxford, Parks Road, Oxford OX1 3PH, United Kingdom; ‡ Walker Department of Mechanical Engineering, The University of Texas at Austin, 204 East Dean Keeton Street, Austin, Texas 78712, United States; § Materials Graduate Program, Texas Materials Institute, The University of Texas at Austin, 204 East Dean Keeton Street, Austin, Texas 78712, United States

**Keywords:** Water-based, Mechanical lithography, Sustainability, Polymer substrates

## Abstract

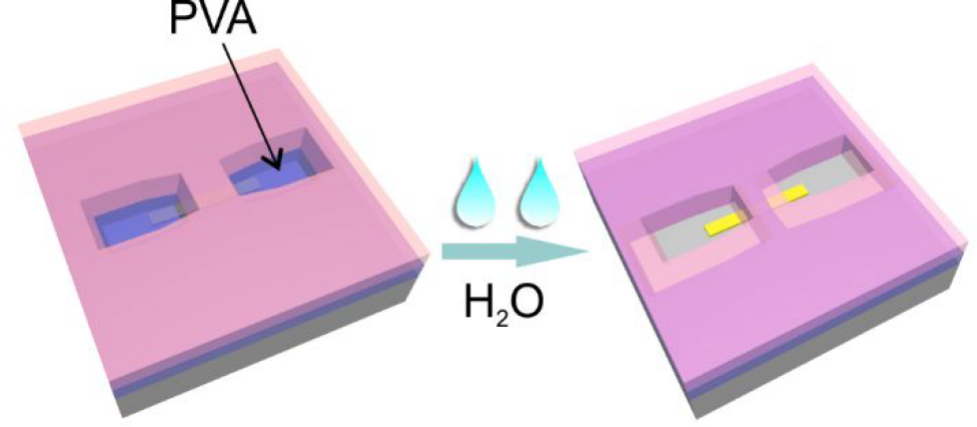

Sustainability has become a critical concern in the semiconductor industry as hazardous wastes released during the manufacturing process of semiconductor devices have an adverse impact on human beings and the environment. The use of hazardous solvents in existing fabrication processes also restricts the use of polymer substrates because of their low chemical resistance to such solvents. Here, we demonstrate an environmentally friendly mechanical, bilayer lithography that uses just water for development and lift-off. We show that we are able to create arbitrary patterns achieving resolution down to 310 nm. We then demonstrate the use of this technique to create functional devices by fabricating a MoS_2_ photodetector on a polyethylene terephthalate (PET) substrate with measured response times down to 42 ms.

## Introduction

Manufacturing technologies in the semiconductor industry utilize a wide range of advanced micro- and nanofabrication technologies for applications in electronics,^1−4^ optoelectronics,^5−8^ microfluidics,^9−11^ health care,^12−15^ and energy conservation.^16−18^ However, over 200 high-purity organic and inorganic chemicals are used for the manufacture of semiconductor devices.^19^ This presents an important challenge of disposing these chemical wastes properly, especially as the majority of them are harmful to human beings as well as the environment. Even before they are disposed of, hazardous chemicals which may contain carcinogens pose a threat to the health of workers in semiconductor factories.^20−23^ Hazardous wastes also have a negative effect on our environment, potentially resulting in air, soil, and water pollution.^24,25^ Thus, environmental sustainability has become a critical concern in the manufacturing of high-tech semiconductor devices. Current manufacturing of high-resolution semiconductor devices primarily relies on photolithography as the patterning technique of choice. During the fabrication of these resist-based lithography techniques, development and lift-off steps utilize alkaline solutions and organic solvents as developers and strippers. These are two of the main sources of hazardous chemical wastes.^26,27^ The U.S. Environmental Protection Agency developed a waste management hierarchy, which states that the most preferred approach is source reduction and reuse, followed by recycling, energy recovery, treatment, and disposal.^28^ Therefore, the development of a water-based manufacturing technique which limits the number of hazardous chemicals used at the source is essential to the minimization of chemical waste. For example, Hong et al. have recently proposed an eco-friendly electron beam lithography method using ice as the resist, which avoids the use of solvents during the fabrication.^29^

Apart from the limitation of using hazardous chemicals, a water-based manufacturing technique which avoids radiation or electron damage could also enable a wider range of materials to be processed as functional devices, such as polymer substrates and two-dimensional (2D) materials. Polymers are easily degraded when they are exposed to strong chemicals or radiation, and radiation damage from high-energy photons or electron beams may introduce defects in 2D materials during the fabrication process.^30,31^ Indeed, most flexible 2D materials-based devices are currently fabricated by transferring prefabricated devices to flexible polymer substrates to avoid such damage.

In this paper, we propose a water-based mechanical bilayer lithography process for development and lift-off. We use two layers of resist where the bottom layer of resist is water-soluble. The goal is to enable water to act as a resist developer. While the environmental advantages of this approach are obvious in that hazardous solvents are avoided, there is also a significant functional benefit. This water-based technique protects polymer substrates from solvent damage and 2D materials from photolithography-induced radiation damage.

As we also demonstrate, another advantage is that the nanoscale tip is not required to be in direct contact with the substrate during patterning. Conventional atomic force microscope (AFM)-based patterning results in either undercutting (i.e., small forces cause the loss of pattern information) or overcutting (i.e., large forces damage both the tip and substrate)^32,33^ and is a challenge as tip forces require very precise and frequently adjusted force control. Such control is difficult on a large scale. In this paper, we demonstrate that our mechanical bilayer lithography is suitable for both SiO_2_/Si and flexible polyethylene terephthalate (PET) substrates. It is capable of writing arbitrary patterns and reaching a resolution of 310 nm with scope for further improvement. As 2D materials are frequently influenced by solvent processing, we then apply this technique to fabricate a flexible MoS_2_ photodetector as a demonstrator and achieve photoresponsivity of 29 mA/W and photoswitching speed of 42 ms under a laser illumination of 633 nm wavelength, showcasing the applicability of this technique to making functional devices.

## Results and Discussion

**Figure 1:**
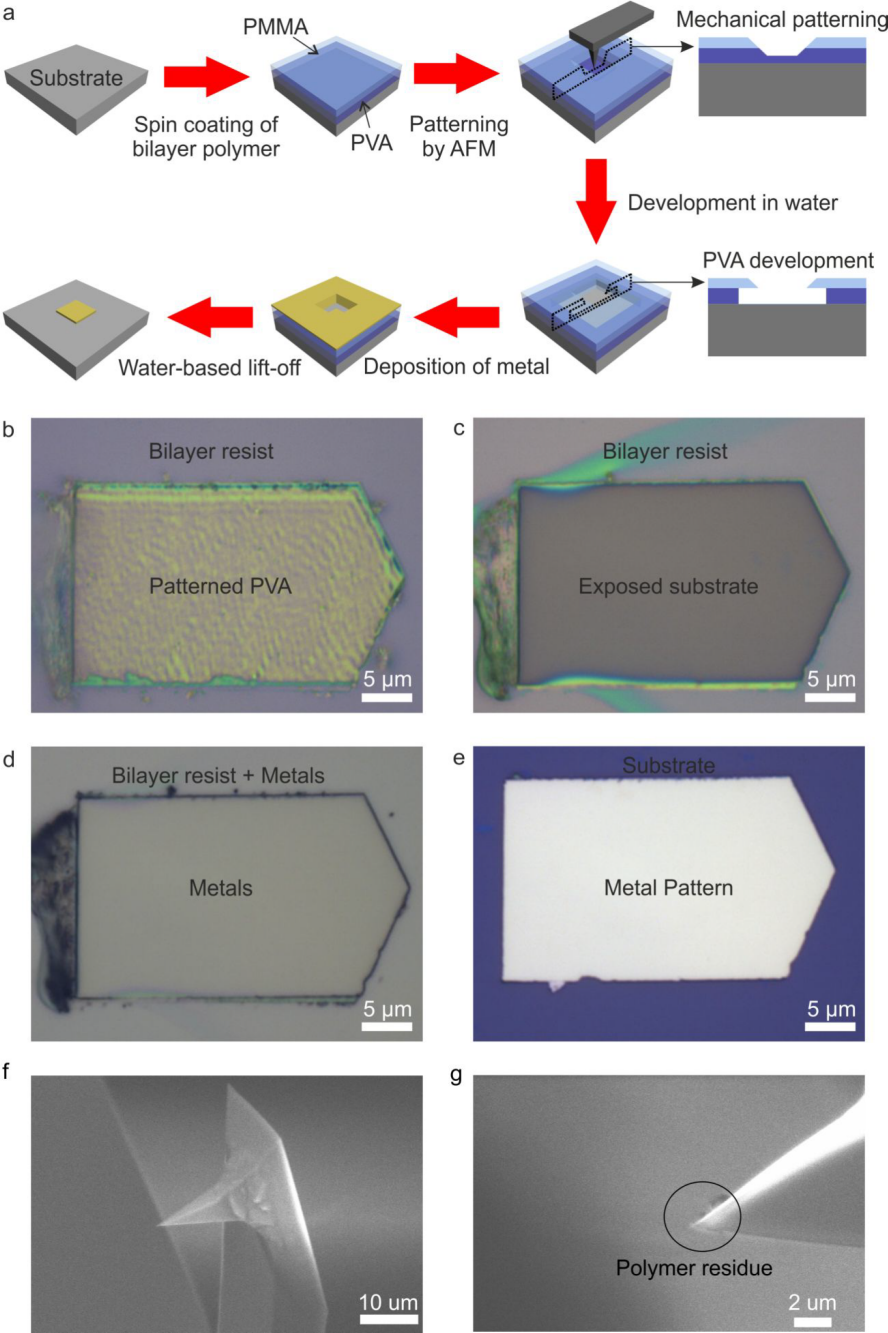
Water-based mechanical bilayer lithography. (a) Schematic process flow of the fabrication process. (b–e) Optical images of an electrode pattern after key steps, including (b) mechanical patterning of the bilayer resist, (c) water-based development, (d) metal deposition prior to lift-off, and (e) metal electrode after lift-off. (f) SEM image of a new tip. (g) SEM image of a used tip.

Figure 1 illustrates our bilayer lithography process. Two layers of resist, a top layer of poly(methyl methacrylate) (PMMA) and a bottom layer of poly(vinyl alcohol) (PVA) were utilized. Patterns were written on the bilayer resist by applying sufficient force to penetrate through to the bottom PVA layer. It is unnecessary to remove the entire PVA layer during patterning as subsequent development can remove the unpenetrated PVA; this enables our process to be more tolerant in the application of force. The pattern in Figure 1b is a combination of a 30 × 20 μm rectangle and a triangle with the longest edge being 20 μm. Here, much of the PVA layer remains, so the surface appears relatively rough compared to that in the unpatterned area, and the motion of the nanoscale tip in the form of straight lines from right to left is observed. We also observe that the removed resist is piled up on the left side of the pattern.

For the development, we use the fact that PVA is soluble in water whereas PMMA is not. Thus, the patterned area allows the water to dissolve the PVA without affecting the PMMA. We found that the rate of development of PVA in room-temperature water to be faster than reasonable timescale achievable. Structural collapse of the bilayer occurs due to the development of the unpatterned PVA at this temperature, resulting in loss of pattern information after lift-off (SI Figure S1). Therefore, we used cold water at 0–5 °C to avoid overdeveloping for timescale of ∼1 s. These parameters are suitable for developing patterns with different dimensions on both SiO_2_/Si and PET substrates.

Following development, the underlying substrate is exposed. The smoothness of the area following this step is shown in Figure 1c, which demonstrates that the roughness of the patterning is not a material issue for the lithography process. This underlines the tolerance of the lithography process to variations of the mechanical force. We then use this pattern to carry out our metal lift-off processes. We define these lift-off patterns by depositing 5 nm Cr and 50 nm Au (Figure 1d) with lift-off carried out by overnight immersion in water. A pattern thus obtained is shown in Figure 1e, validating our approach.

Another advantage of our technique is the flexibility to control the mechanical force applied on the AFM tip. Single-layer resist used in conventional AFM mechanical lithography requires precise control of the mechanical force to avoid overcutting and undercutting problems. In our process, however, we write patterns in the PVA layer without the need to remove the entire PVA layer. Thus, the mechanical force applied can be flexibly selected within a range, which allows for good error tolerance.

To investigate this, we used gradually increasing mechanical forces and measured the corresponding depths after patterning. The force versus depth curve (SI Figure S2) shows that the best mechanical forces can be selected in a relatively wide range from 10 to 15 μN. In our measurements, the thicknesses of the PMMA and PVA layers are both about 185 nm. For forces <10 μN, the tip does not penetrate the PMMA layer. For forces >10 μN, the tip cuts through PMMA layer and begins to mechanically pattern the PVA layer. When the mechanical force reaches 15 μN, the tip starts to interact with the SiO_2_/Si substrate. Thus, for a given thickness of PMMA and PVA, the applied force can be flexibly selected (ranging from 10 μN to 15 μN in this case), allowing for robust patterning over a large area. In addition, the tip is controlled to mechanically pattern the PVA layer without contacting the substrate, which greatly reduces the wearing damage to the tip. Figure 1f,g compares a new and a used tip. The used tip is still sharp, although some polymer residues remain around the tip after patterning as seen using scanning electron microscopy (SEM). We also find that these residues are readily removed in acetone and water and the tip can be reused to do patterning.

**Figure 2:**
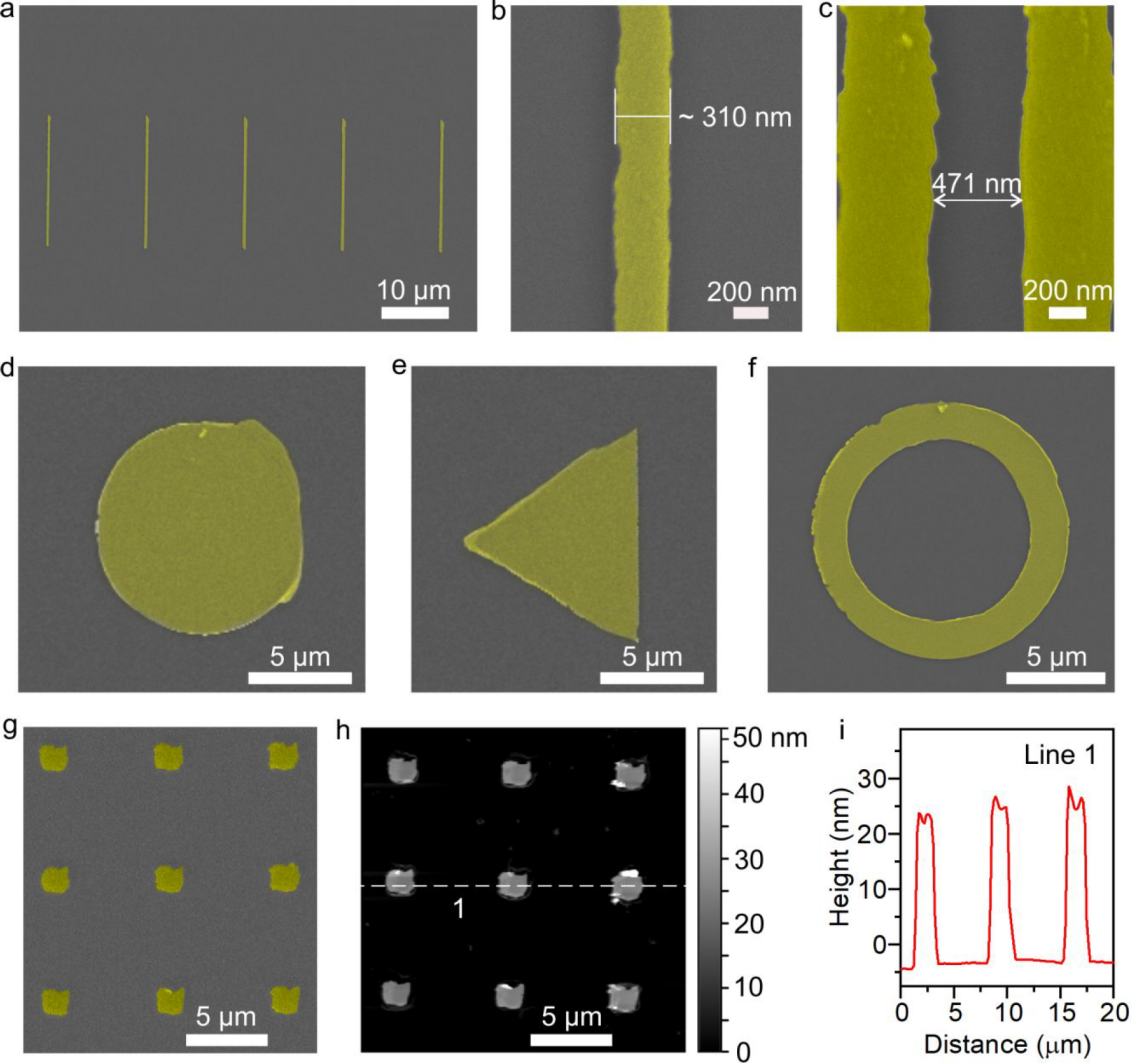
Patterns on SiO_2_/Si substrates fabricated by bilayer lithography. (a–f) False-colored SEM micrographs of (a) line patterns, (b) highest resolution line pattern, demonstrating an achievable line width of 310 nm, (c) spacing of 471 nm between two patterns, (d) a circular pattern, (e) a triangular pattern, and (f) a ring pattern. (g) SEM image of nine square patterns. (h) AFM scan of these square patterns. (i) Height profile of the dashed line marked in (h).

We then investigate the use of this technique on Si substrates with SiO_2_ capping. Figure 2a shows an array of 20 μm long line patterns that was patterned onto SiO_2_/Si substrates. We obtain line widths as small as 310 nm as shown in Figure 2b, using our technique without any observable resist residue around the line pattern. The patterning width is mainly determined by the tip size.^34,35^ Thus, it is possible to obtain higher resolution by using sharper tips and optimizing other parameters such as tip geometry and composition, development time and temperature, and mechanical force. Figure 2c shows the minimum spacing between two patterns using our technique to be 471 nm, indicating excellent line-spacing capability in addition to the high resolution. To demonstrate the adaptability of our technique to create arbitrary microscale patterns, we patterned a circle, a triangle and a ring using this technique on SiO_2_/Si substrates. We then deposited 5 nm Cr and 20 nm Au, followed by lift-off. SEM images of a circle with a radius of 5 μm, an equilateral triangle with sides of 10 μm, and a ring with the two radii differing by 2 μm are shown in Figure 2d–f, respectively, demonstrating that this technique can also fabricate structures with different shapes.

Figure 2g presents a further array of square patterns with an average side length of 1.8 μm, formed by the deposition of 10 nm Cr and 20 nm Au. The topography of each pattern was characterized by AFM after lift-off. The average thickness of all square patterns is around 30 nm, which was consistent with the deposition parameters (Figure 2h,i). The variation of the thickness in each pattern is within 5 nm. This indicates that bilayer lithography can achieve uniform surface topography of multiple patterns.

We then investigate the suitability of our patterning technique on flexible substrates. Given that our mechanical lithography can accommodate changes to substrate topography, combined with the force tolerance of our technique as described previously, this technique is well suited to flexible substrates. Combined with the potential to eschew solvent use during development, it would allow one to use flexible substrates such as PET that are not compatible with hazardous processes.

We coated PVA and PMMA layers on PET substrates and patterned an array of square patterns into the bilayer (SI Figure S4c). To demonstrate the flexibility of controlling mechanical forces applied on the tip, we applied mechanical forces of 10, 11, and 12 μN from the left to the right column sequentially during patterning. The depth profile shows that the average depth is ∼216 nm (SI Figure S4a,b). The measured depths prior to development do not increase with the increasing applied force, which is attributable to resist residue around the tip during patterning, leading to less precise depth profiles.

Deposition of metals on PET substrates is different from SiO_2_/Si substrates because of the poor adhesion between metals and polymers caused by their low wettability.^36^ To improve their adhesion, we treated the substrate with O_2_ plasma before the deposition of 5 nm Cr and 20 nm Au. After lift-off, all patterns were obtained even though different mechanical forces were used for each column, underlining the robustness of our technique to mechanical force.

**Figure 3:**
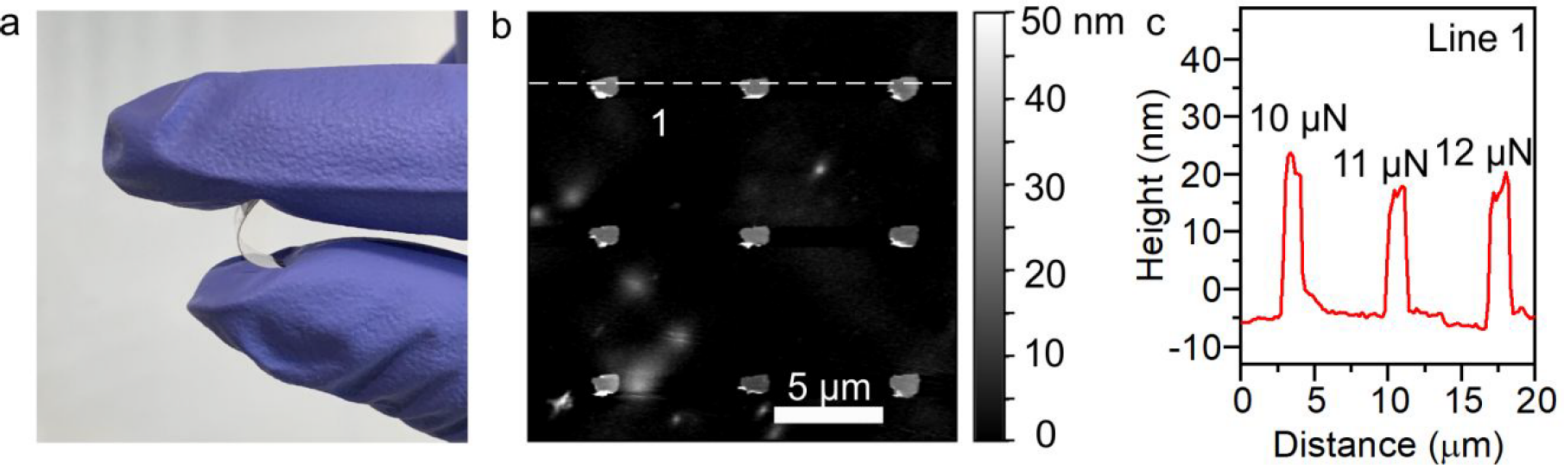
Square patterns on PET substrates. (a) A photo of a PET substrate with square patterns of Cr and Au. (b) AFM scan of these square patterns on the PET substrate. (c) Height profile of the dashed line marked in (b).

Figure 3a shows the PET substrate onto which the square patterns were deposited. The height profiles indicate that the mean length of each side of the squares is 0.93 μm and their mean thickness of each pattern is 25 nm, which is in agreement with the deposition parameters (Figure 3b,c). The thickness at the edge of the patterns is slightly higher than 25 nm because of the high roughness of PET substrates, which results in a worse lift-off than that on the smoother SiO_2_/Si substrates; this could be improved in future experiments via the use of ultrasmooth substrates. Regardless, the technique works very well on flexible substrates.

**Figure 4:**
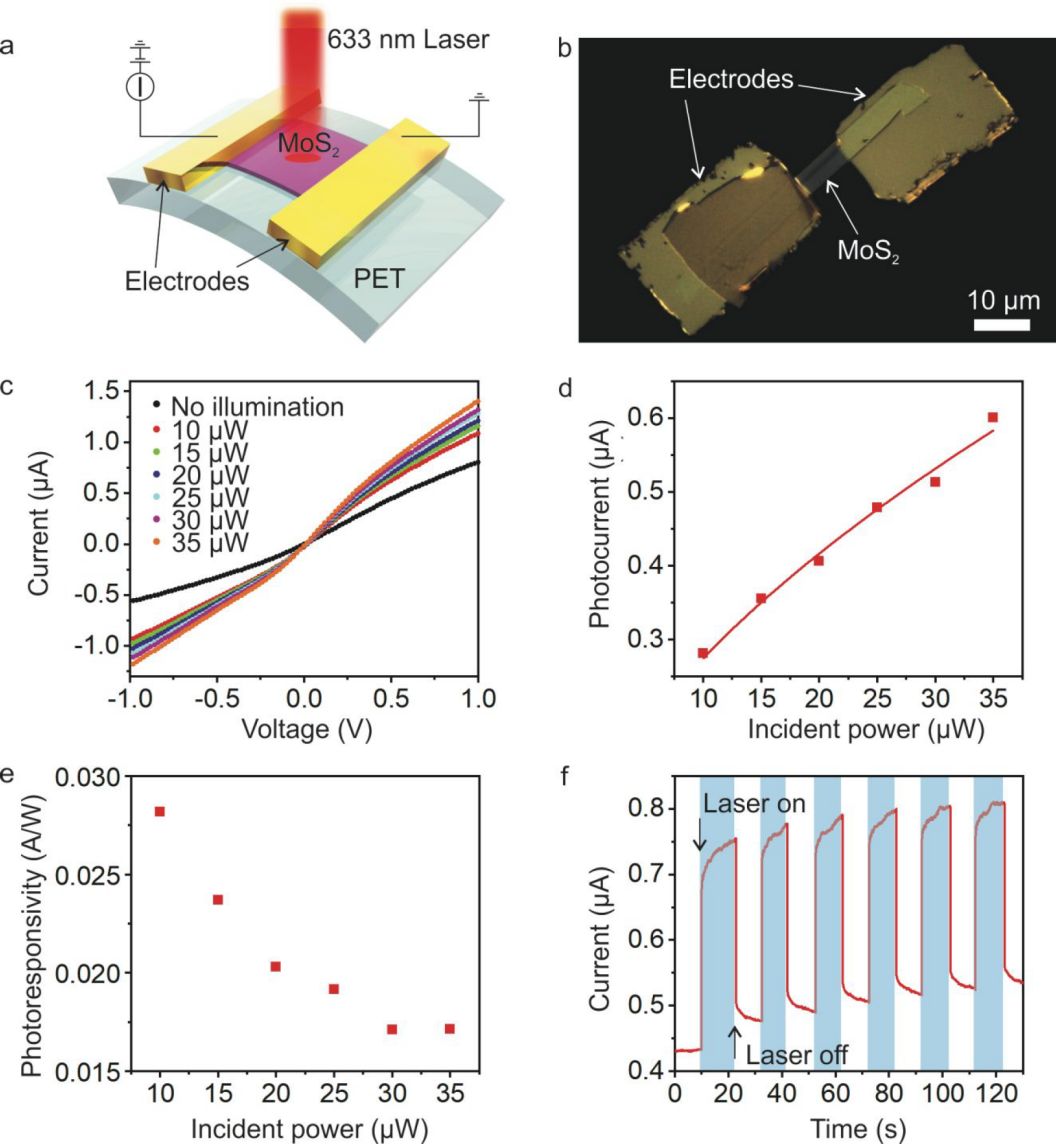
Flexible MoS_2_ photodetector device. (a) Schematic diagram of the flexible MoS_2_ photodetector. (b) Optical image of the flexible MoS_2_ photodetector. (c) *I*–*V* curve of the photodetector in dark and under different illumination powers. (d) Photocurrent of the photodetector under different illumination powers for *V* = 1 V. (e) Photoresponsivity of the MoS_2_ photodetector. (f) Time-resolved photoresponse of the photodetector for *V* = 0.5 V and an illumination power of 38 μW.

To further explore practical applications of water-based bilayer lithography, we used this technique to fabricate a flexible MoS_2_ photodetector on the PET substrate. A multilayer film of MoS_2_ with thickness of 78 nm was obtained by mechanically exfoliating bulk MoS_2_ and then transferring to a PET substrate (SI Figure S5a,d). Bilayer lithography was subsequently used to pattern Au electrodes onto the MoS_2_ (Figure 4a,b).

The fabricated flexible MoS_2_ photodetector device was characterized by measuring the current through the MoS_2_ channel under dark conditions and using a 633 nm laser source with illumination powers ranging from 10 to 35 μW (Figure 4a). The current shows a linear relationship with small voltages *V* supplied to photodetector, indicating an ohmic response (Figure 4c). The device also demonstrates a clear response to 633 nm laser. As can be seen from Figure 4d, the photocurrent at *V* = 1 V exhibits a sublinear increase with increasing incident power.

The photoresponsivity is defined as *R* = *I*_ph_/*P*_light_, where *I*_ph_ is the photocurrent and *P*_light_ is the incident light power. Figure 4e shows the photoresponsivity under various incident light powers from 10 to 35 μW acquired at the supplied voltage of *V* = 1 V. The photoresponsivity and incident light power exhibit negative correlation (Figure 4e). This is mainly due to the increase in photogenerated carriers under high incident light power. The highest photoresponsivity measured was 29 mA/W at an incident light power of 10 μW.

The time-resolved photoresponse was further investigated by switching the illumination on and off. The current through the MoS_2_ channel current jumps to a high value when the illumination is turned on and recovers immediately to near the dark current level when the illumination is turned off (Figure 4f). The calculated response time of the MoS_2_ photodetector is about 42 ms, which compares favorably with those fabricated using photolithography (SI Figure S8) as well as from published values.^6,37,38^ We attribute this to the reduction of surface trap states on the MoS_2_ channel, further demonstrating the advantages of using our environmental-friendly bilayer lithography method. We also noted an overall trend of increasing dark current during our measurement mainly due to the laser-induced heating effects.^39^ As the thermal conductivity of PET is very low (about 0.15 W m^–1^ K^–1^) compared to that of other silicon substrates such as SiO_2_ (about 1.3 W m^–1^ K^–1^), the heating effects become more prominent for MoS_2_ photodetectors on PET substrates.^39^

## Conclusions

We have demonstrated a water-based mechanical lithography approach that exploits a bilayer resist structure. Our water-based fabrication approach is suitable for patterning on both SiO_2_/Si and PET substrates. The use of water for development and lift-off helps to minimize the chemical wastes at the source, leading to a more sustainable technique. Crucially, this also adds process functionality because the technique enables protection of polymer substrates from solvent-based damage. Our technique can achieve arbitrary patterns with a demonstrated line resolution down to 310 nm. There is significant scope to improve this using sharper probes, and further optimization of parameters such as tip geometry, tip material, development time, water temperature, and mechanical force would significantly improve achievable resolution and quality. Further investigation into the highest achievable aspect ratios is required to understand the potential applicability of this technique. The technique has potential for fabricating novel functional devices that were previously not possible such as our flexible MoS_2_ photodetector and has the potential to be an important process toolkit that can help overcome some of the environmental costs of semiconductor fabrication.

## Materials and Methods

### Spin Coating of PVA and PMMA Layer

For patterning on SiO_2_/Si substrates and PET substrates, A4 PMMA (MW 495k from MicroChem) in anisole was used for spin coating. A1 PMMA (MW 495k from MicroChem) in anisole was used to pattern electrodes for the MoS_2_ photodetector. PVA solution (5% w/v) was prepared by dissolving PVA (Mowiol 4–88, MW ∼ 31 000 from Sigma-Aldrich) in DI water. A layer of PVA was coated on the SiO_2_/Si substrate for 30 s at 2000 rpm and heated for 10 min at 150 °C. A layer of PMMA was then coated on the SiO_2_/Si substrate for 60 s at 4000 rpm and heated for 10 min at 150 °C. Because of the low glass transition temperature of PET substrates, heating time and temperature after coating PMMA and PVA on PET substrates were adjusted to 70 °C and 20 min.

### Patterning and Development

All patterns were designed by AutoCAD software and loaded in to an AFM (Asylum MFP-3D) before patterning. The patterning was carried out by maintaining a constant cantilever deflection. Tap300-G probes (*k* = 40 N/m, Budget Sensors) with rotated tips (radius <10 nm) were used for both AFM imaging and patterning. To pattern lines with nanoscale features, the tip scanned the pattern 2–3 times. Following patterning, DI water at a temperature of 0–5 °C was used for the development.

### Deposition and Lift-off

A thin film of Cr/Au was thermally evaporated on substrates after the development. Then DI water was used for lift-off overnight.

### Preparation of MoS_2_

A bulk MoS_2_ crystal (Ossila) was mechanically exfoliated into a thin film of MoS_2_ by Scotch-tape cleavage approach and was subsequently transferred onto PET substrates using polydimethylsiloxane (PDMS) stamp.

### Characterization and Device Measurement

Patterns were characterized by Nikon optical microscope Eclipse LV100ND and ZEISS Merlin SEM. Topographies of patterns were analyzed by Asylum MFP-3D AFM. Electrical measurements were carried out using a Keithley source meter 2614B under room temperature in ambient conditions. A 633 nm laser diode (Thorlabs, LP633-SF50, spot size ∼10 μm) was used as the illumination source. The power of the illumination was measured by a power meter (Thorlabs PM100D) before each electrical measurement.

## Supporting Information Available

Comparison between proper development and improper development, investigations of mechanical forces applied on the tip, square patterns on SiO_2_/Si or PET substrates at different stages during the fabrication process, Raman spectroscopy and topography of MoS_2_ thin film, cross section of the bilayer structure after development, additional data of MoS_2_ photodetector fabricated by bilayer lithography, and a comparison with flexible MoS_2_ photodetector fabricated by photolithography (PDF)
