# Supplementary material for: Nanoscale Bilayer Mechanical Lithography Using Water as Developer
Source: Nano Lett. 2021 Apr 22;21(9):3827–34. doi: 10.1021/acs.nanolett.1c00251 (PMC8289280; doi:10.1021/acs.nanolett.1c00251)
Supplement: Supplementary file 1 — nl1c00251_si_001.pdf [file nl1c00251_si_001.pdf]

Nanoscale Bilayer Mechanical Lithography Using Water as Developer

Yu Shu[1], Benjamin F. Porter[1], Eugene J.H. Soh[1], Nikolaos Farmakidis[1], Seongdong Lim[1], Yang Lu[1], Jamie H. Warner[2,3], Harish Bhaskaran[1*]

[1]Department of Materials, University of Oxford, Parks Road, Oxford OX1 3PH, UK.
[2]Walker Department of Mechanical Engineering, The University of Texas at Austin, 204 East Dean Keeton Street, Austin, Texas, 78712, United States
[3]Materials Graduate Program, Texas Materials Institute, The University of Texas at Austin, 204 East Dean Keeton Street, Austin, Texas, 78712, United States
[*]Corresponding authors: E-mail: harish.bhaskaran@materials.ox.ac.uk

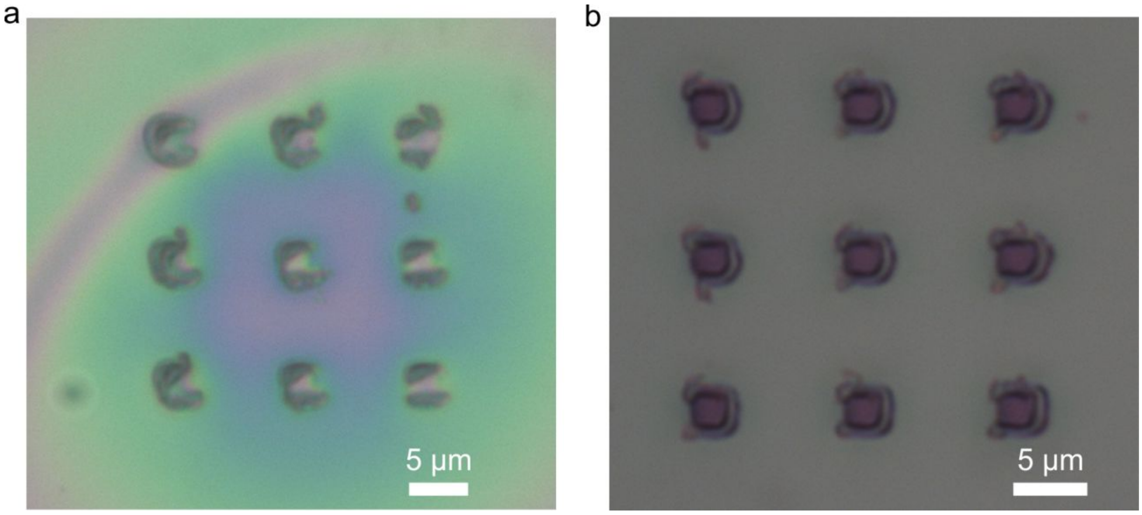

Figure S1 Comparison between proper development and improper development. a. Optical image of square patterns after improper development. Structural collapse occurred due to the development of unpatterned PVA. **b**. Optical image of square patterns after proper development.

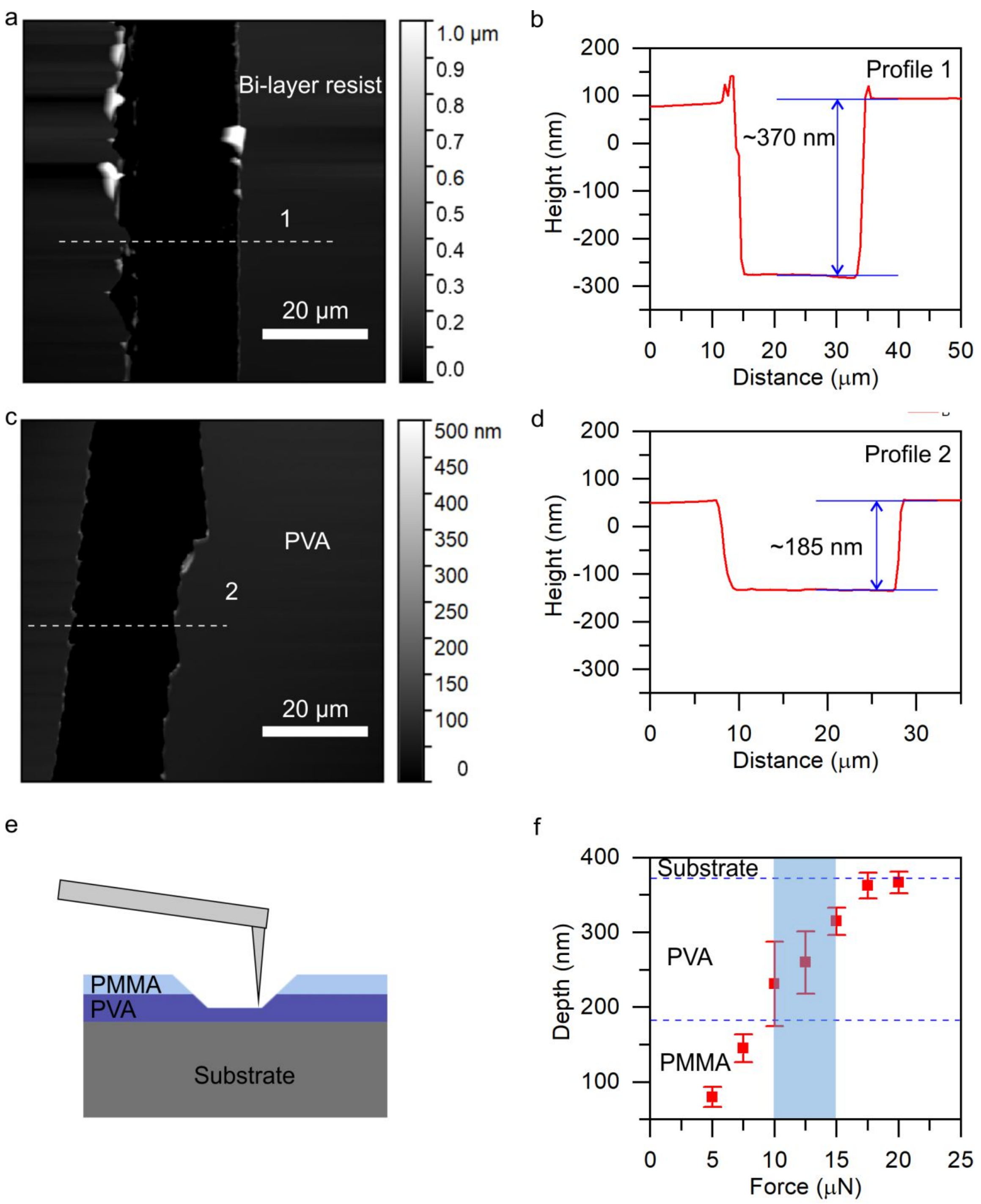

Figure S2 Investigation of mechanical forces that should be applied on the AFM tip. **a**. AFM image of bilayer resist with a gap. **b**. Height profile of the dashed line 1 in **a**. **c**. AFM image of PVA with a gap. **d**. Height profile of the dashed line 2 in **c**. **e**. Schematic diagram of the AFM tip patterning. **f**. Force vs depth curve obtained by using gradually increasing mechanical forces and measuring the corresponding depth after patterning.

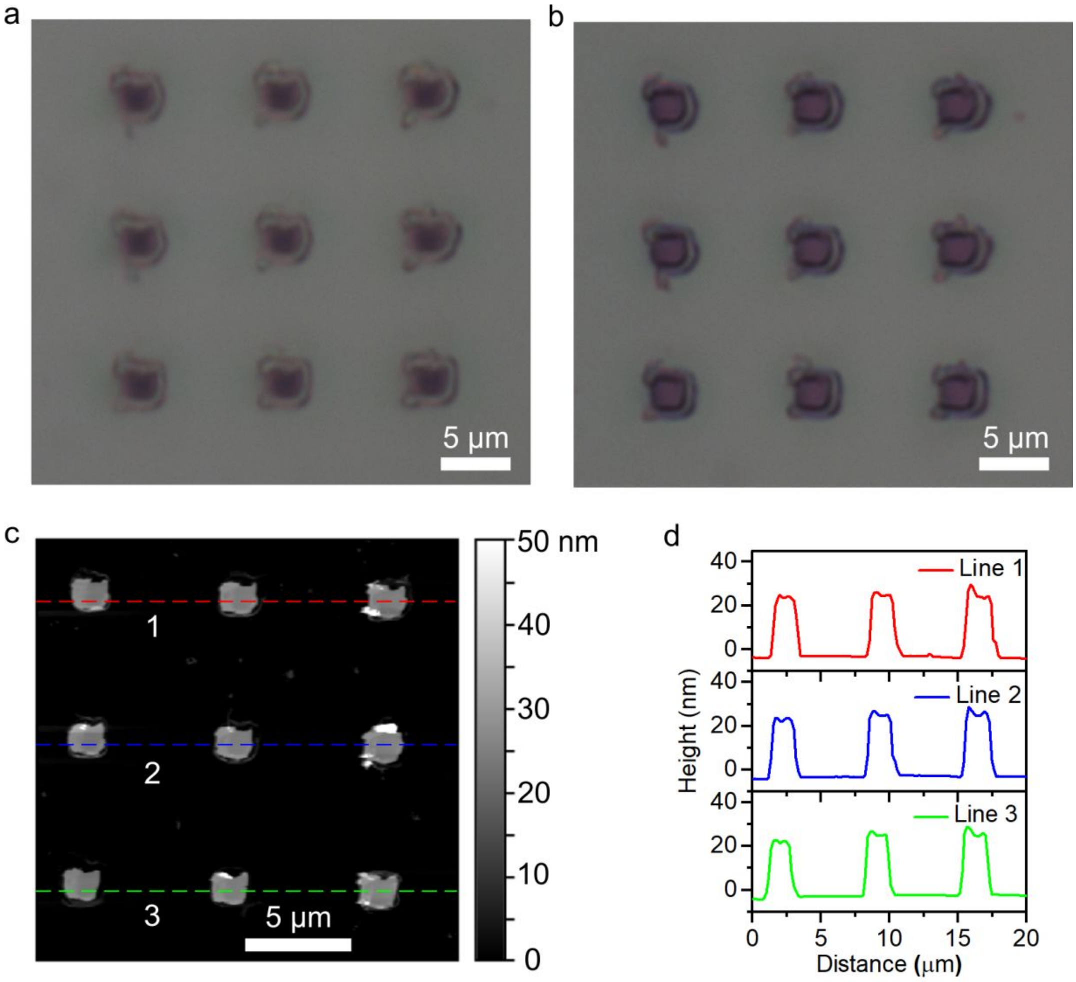

Figure S3 Square patterns on SiO_2/Si substrates at different stages of the fabrication process. **a**. Optical image of square patterns after mechanical patterning. **b**. Optical image of square patterns after development in water. **c**. AFM image of square patterns after lift-off. **d**. Height profiles of the dashed lines in c.

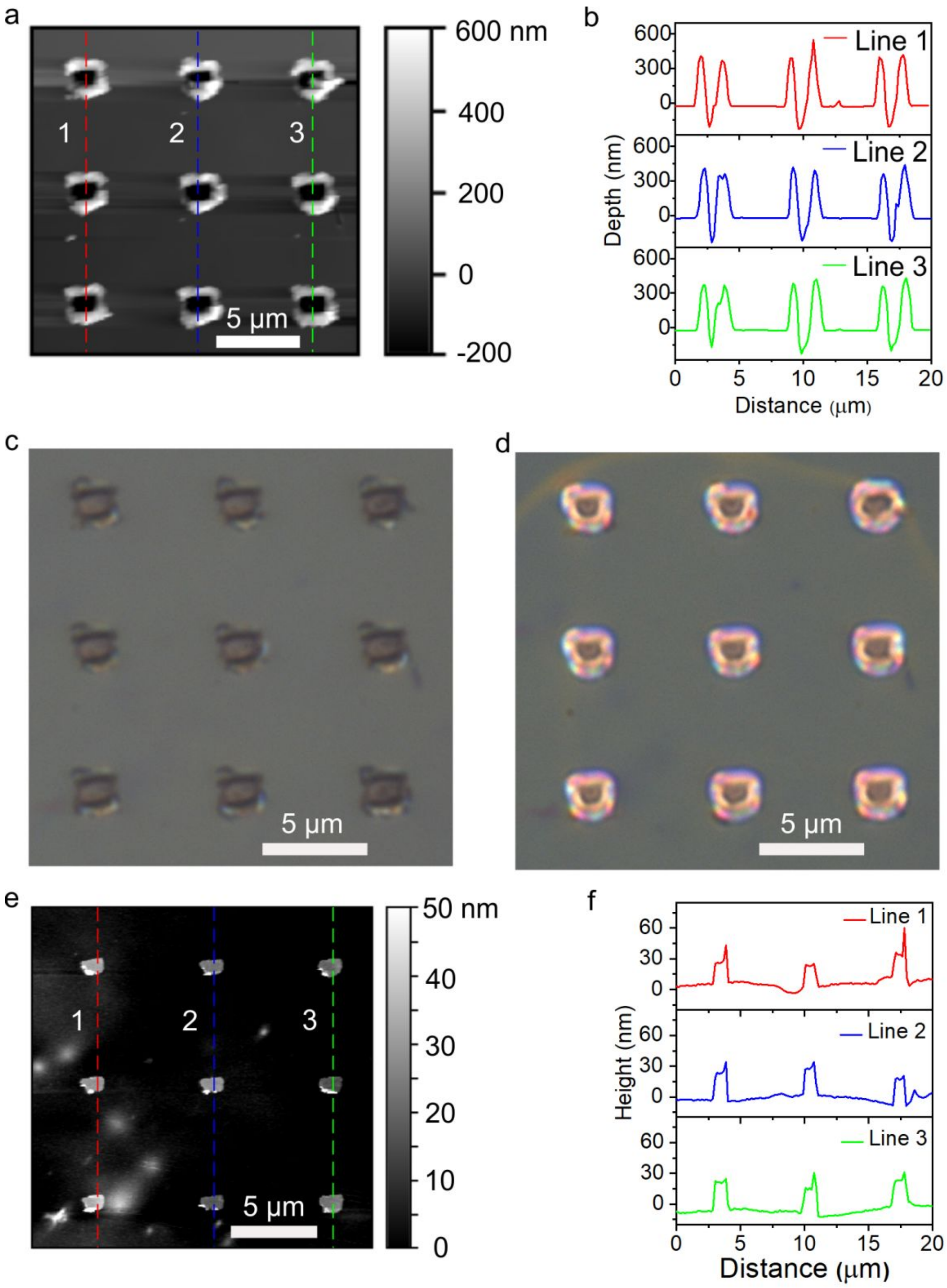

Figure S4 Square patterns on PET substrates at different stages of the fabrication process. a. AFM image of square patterns after patterning. **b**. Height profiles of the dashed lines in **a**. **c**. Optical image of square patterns after patterning. **d**. Optical image of square patterns after development in water. **e**. AFM image of square patterns after lift-off. **f**. Height profiles of the dashed lines in **e**.

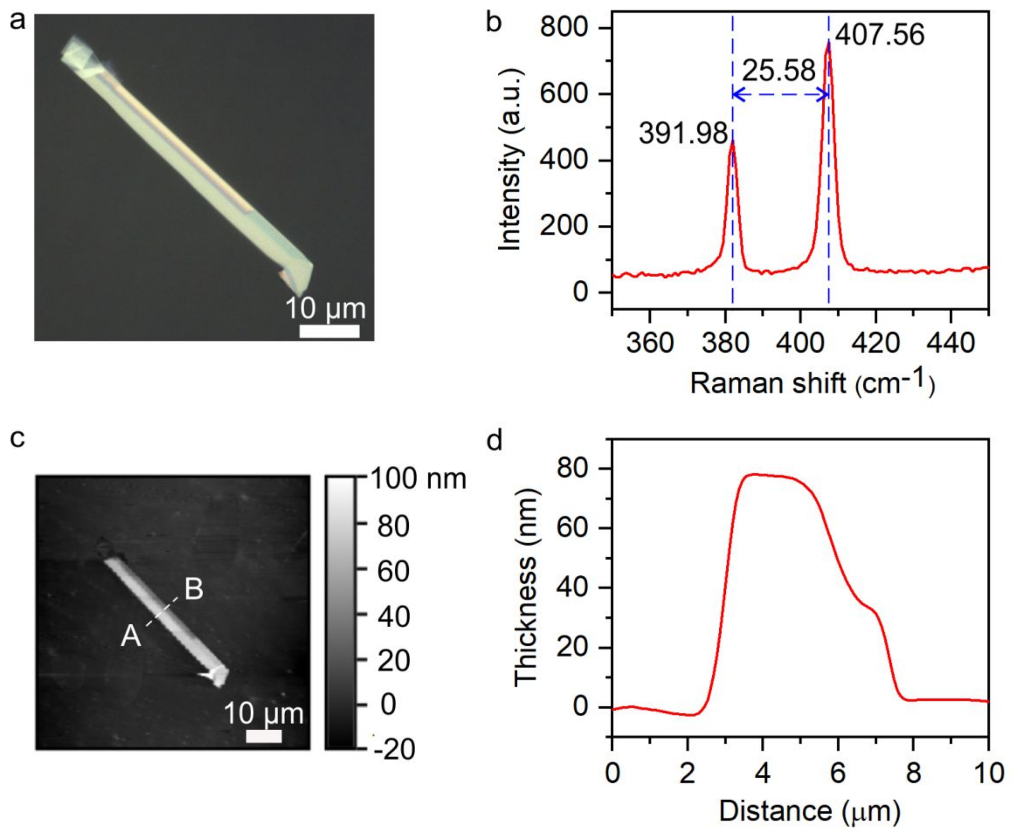

Figure S5 MoS_2 thin film transferred onto the PET substrate. a. Optical image of MoS_2 thin film transferred on the PET substrate before patterning. **b**. Raman spectroscopy of MoS_2 thin film on the PET substrate. **c**. AFM image of MoS_2 thin film on the PET substrate. **d**. Height profile of the dashed line AB in **c**.

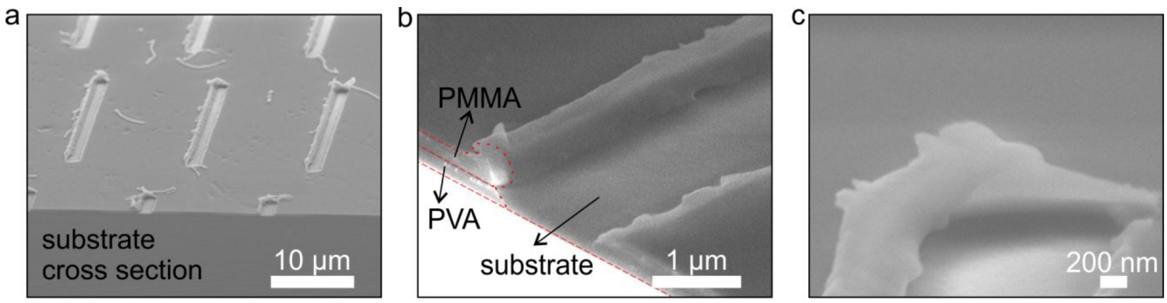

Figure S6 Cross section of bilayer structure after development. a. SEM image of the cross section under lower magnification. **b**. SEM image of the cross section under higher magnification. **c**. SEM image of the pattern which shows the overhanging structure.

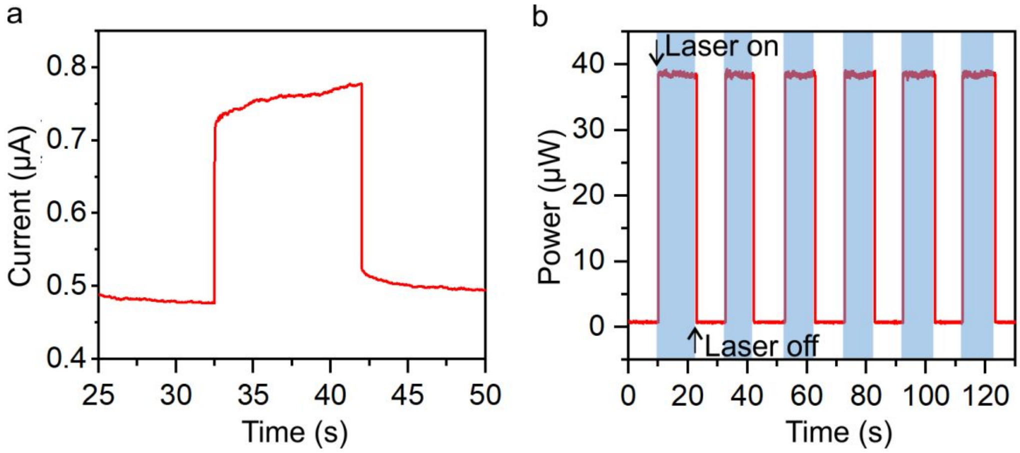

Figure S7 Time-resolved photoresponse of the photodetector under V_{ds} = 0.5 V and the illumination power of 38 μW. a. Expanded view of the time-resolved photoresponse of the photodetector. **b**. Real-time power of the laser at the wavelength of 633 nm.

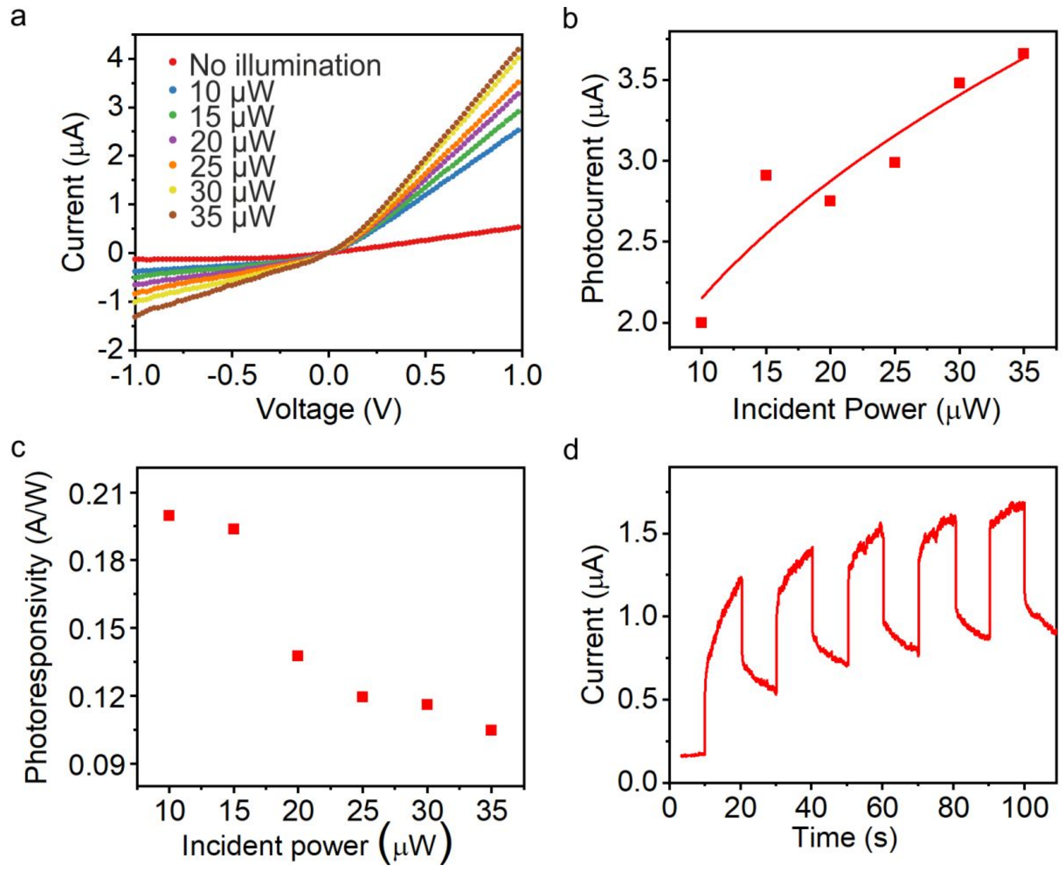

Figure S8 A comparison of photoresponse of the MoS_2 photodetector fabricated by photolithography. **a**. *I-V curve* of the photodetector in dark and under different illumination powers. **b.** Photocurrent of the photodetector under different illumination powers for V = 1 V. **c**. Photoresponsivity of the MoS_2 photodetector. **d**. Time resolved photoresponse of the photodetector for V = 0.5 V and an illumination power of 38 μW. The calculated response time is 348 ms.
